# Computer-aided characterization of the arrhythmogenic substrate after myocardial infarction

**DOI:** 10.1093/europace/euag003

**Published:** 2026-01-09

**Authors:** Manon Kloosterman, Karin C Smits, Job Stoks, Machteld J Boonstra, Veronique M F Meijborg, Pranav Bhagirath, Rachel M A ter Bekke, Joël M H Karel, Marco J W Götte, Peter Loh, Jason D Bayer, Uyên Châu Nguyên, Ruben Coronel, Matthijs J M Cluitmans

**Affiliations:** Department of Cardiology, University Medical Center Utrecht, Utrecht, The Netherlands; Department of Physiology, Maastricht University Medical Center+, Cardiovascular Research Institute Maastricht, Maastricht, The Netherlands; Department of Cardiology, Maastricht University Medical Center+, Cardiovascular Research Institute Maastricht, Maastricht, The Netherlands; Department of Cardiology, Amsterdam Cardiovascular Sciences, Amsterdam University Medical Center, University of Amsterdam, Amsterdam, The Netherlands; Department of Cardiology, University Medical Center Utrecht, Utrecht, The Netherlands; Department of Medical Physiology, University Medical Center Utrecht, Utrecht, The Netherlands; Department of Cardiology, Amsterdam Cardiovascular Sciences, Amsterdam University Medical Center, University of Amsterdam, Amsterdam, The Netherlands; Department of Cardiology, Maastricht University Medical Center+, Cardiovascular Research Institute Maastricht, Maastricht, The Netherlands; Department of Advanced Computing Sciences, Maastricht University, Maastricht, The Netherlands; Department of Cardiology, Amsterdam Cardiovascular Sciences, Amsterdam University Medical Center, University of Amsterdam, Amsterdam, The Netherlands; Department of Cardiology, University Medical Center Utrecht, Utrecht, The Netherlands; Institut de Mathématiques de Bordeaux, UMR 5251, IHU Liryc and University of Bordeaux, Talence 33400, France; Department of Cardiology, Maastricht University Medical Center+, Cardiovascular Research Institute Maastricht, Maastricht, The Netherlands; Department of Experimental Cardiology, Amsterdam University Medical Center, University of Amsterdam, Amsterdam, The Netherlands; Department of Cardiology, Maastricht University Medical Center+, Cardiovascular Research Institute Maastricht, Maastricht, The Netherlands

**Keywords:** Arrhythmias, Imaging, Electrocardiography, Computer simulation

## Abstract

Ventricular tachycardia (VT) and ventricular fibrillation remain major contributors to sudden cardiac death, with current therapies limited by our incomplete understanding of the arrhythmogenic substrate. This narrative review explores recent developments in computer-aided techniques for characterizing the arrhythmogenic substrate, focusing on post-myocardial infarction VT. High-resolution cardiac imaging now enables detailed visualization of structural abnormalities, including heterogeneous scar architecture and fatty infiltration. Sophisticated invasive mapping techniques provide insights into local electrophysiological properties, while novel non-invasive mapping approaches offer complementary views of global electrical patterns. Integration of these modalities through computational simulations allows for mechanistic insights into arrhythmia initiation and maintenance, particularly in post-myocardial infarction VT, where structural and functional substrates interact in complex ways. Emerging artificial intelligence applications enhance substrate analysis through automated feature extraction and pattern recognition, enabling more sophisticated risk stratification. These computer-aided approaches are advancing from research tools to clinical applications, with early evidence suggesting improved ablation outcomes and better risk prediction. However, significant challenges remain in validation, standardization, and clinical implementation of these innovations. This narrative review highlights recent methodological advances and clinical applications of computer-aided substrate characterization, and conceptualizes future directions towards personalized arrhythmia management, also beyond post-infarction VTs.

## Graphical Abstract

**Graphical Abstract:**
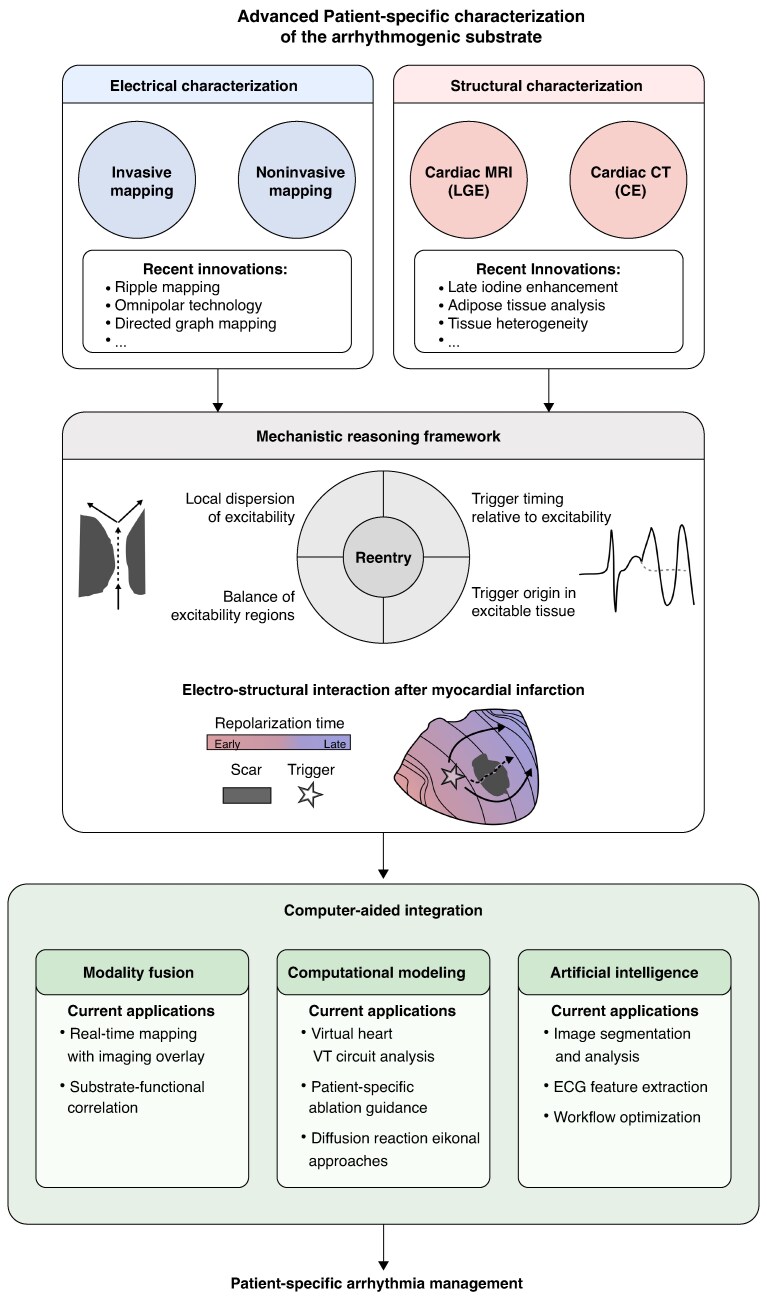

## Introduction

Ventricular tachycardia (VT) and ventricular fibrillation (VF), the primary mechanisms behind sudden cardiac death, are significant contributors to the global burden of cardiovascular diseases. Despite advancements in cardiovascular medicine, the prevalence and mortality associated with these arrhythmias remain substantial, underscoring the need for a deeper understanding of their mechanisms and for more effective management strategies.^1^ This requires patient-specific characterization of the arrhythmogenic substrate. In patients with myocardial infarction (MI), understanding the full characteristics of the VT substrate and its link to arrhythmogenic triggers is essential for improvement of treatment outcomes.^2^ Recent technological advancements in cardiac mapping, imaging, and electrocardiography revolutionized detailed visualization of anatomical, structural, electrical, and mechanical abnormalities that predispose to re-entrant circuits and focal triggers of VT and VF. Techniques such as high-density catheter mapping, non-invasive electrocardiographic imaging (ECGI), late gadolinium enhancement magnetic resonance imaging (LGE-MRI), and contrast-enhanced computed tomography (CE-CT) have provided higher-resolution data and insights into the electrical and structural substrates underlying arrhythmias.

**Figure 1:**
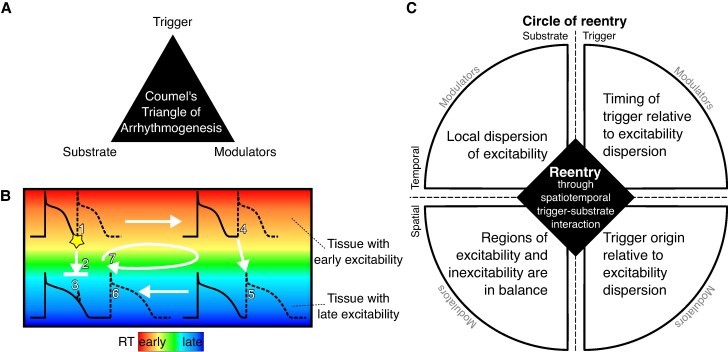
Conceptual frameworks for re-entry initiation. (*A*) The traditional triangle of arrhythmogenesis by Coumel identifies three major factors for arrhythmia initiation. (*B*) Schematic illustration of re-entry in tissue with early and late repolarization time (RT) due to short or long action potential duration, respectively. A premature beat coming from an early RT region (1) may block against tissue that has a longer RT and is still refractory (2) and cannot induce activation in the late RT region (3). If the early RT region is large enough, the activation wave may propagate through that region (4) in a time span that allows the late RT region to repolarize and become excitable, allowing re-activation of the tissue (5). The wave may then travel back to the region that was still refractory but which has become excitable in the meantime (6). By that moment, the tissue from which the premature beat originated has become excitable again, and the wavefront from the late RT region will propagate to that tissue, restarting this circuit (7). (*C*) The Circle of Re-entry extends the triangle of Coumel and proposes four requirements for re-entry arising from spatiotemporal interactions between trigger and substrate: (i) local dispersion of excitability (e.g. steep RT gradients), (ii) a balance in size of the region of excitability and the region of inexcitability (e.g. sufficiently large region of early RT), (iii) a trigger originating at a time when some tissue is excitable and other tissue is inexcitable (e.g. an early premature beat), and (iv) which occurs from an excitable region (e.g. from early RT region). Each of the four elements can be affected by modulators. Obtained from Cluitmans et al.^4^

These technological advances come together with new conceptual insights. Historically, the study of arrhythmogenic mechanisms has evolved from simplistic models to a sophisticated understanding incorporating the intricate interplay between cardiac anatomy, electrophysiology, genetics, and modulators. The Triangle of Coumel^3^ and the more recent circle of re-entry concept^4^ have provided frameworks to understand the multifactorial nature of arrhythmogenesis (*Figure 1*). Additionally, advances in biophysical and computational modelling have significantly enhanced the interpretative power of advanced cardiac imaging and mapping, offering insights into the complex electrophysiological dynamics underlying arrhythmias.^5^ By integrating anatomical and electrical data into patient-specific models, these computational approaches reveal the individual mechanisms of arrhythmogenesis, including electrical impulse propagation and the formation of re-entrant circuits.^6,7^ This synergy between clinical data and computational modelling represents a leap towards precision medicine in treating ventricular arrhythmias, optimizing patient care through tailored therapeutic strategies. Additionally, artificial intelligence (AI) has emerged as a potentially powerful tool, capable of analysing complex datasets to uncover novel characteristics of the arrhythmogenic substrate.^8,9^ AI’s ability to identify patterns within high-dimensional data could further facilitate the development of predictive models for arrhythmia risk stratification and personalized treatment strategies.

This narrative review examines how computer-aided techniques are advancing our understanding and characterization of the arrhythmogenic substrate. We focus on post-MI VT as a model where structural and functional abnormalities interact in well-documented but complex ways.^10,11^ We first examine advances in structural characterization through cardiac imaging, followed by innovations in electrical mapping, both invasive and non-invasive. We then explore how computational approaches enable integration of these modalities and provide mechanistic insights. Finally, we discuss emerging applications of AI and consider challenges and future directions for the field.

## Characteristics of arrhythmogenic substrate

A combination of a trigger and arrhythmogenic substrate can result in the occurrence of re-entry and VT/VF.^4^ Trigger often refers to focal activity resulting from abnormal impulse formation like early or delayed afterdepolarizations. Substrate refers to pre-existing functional (i.e. electrophysiological) or structural abnormalities. Functional substrates can be dynamic, influenced by factors such as intrinsic beat-to-beat variations, autonomic tone, metabolic states, or drug effects. Structural substrates, on the other hand, are often the result of anatomical changes such as scar tissue, fibrosis, or fat infiltrations.^12^ A classic example of trigger-substrate interaction is the R-on-T phenomenon, where a premature ventricular contraction (PVC; the ‘R’) falls on the vulnerable phase of the myocardial recovery (the ‘T’), leading to re-entrant activation. Here, the timing of a trigger interacts with a substrate that is still partially recovering, making it susceptible to arrhythmias.

To understand arrhythmogenesis, concepts such as the Triangle of Coumel and the Circle of Re-entry provide valuable frameworks (*Figure 1*). The triangle of Coumel helps in understanding the interplay between the triggering event, the arrhythmogenic substrate, and modulating factors.^3^ The Circle of Re-entry extends this concept by dissecting the spatial and temporal characteristics essential for re-entry to occur.^4^ It emphasizes the importance of local dispersion of excitability in time and space, such as steep repolarization time gradients or fibrosis. A critical aspect of this framework is the balance between areas of early and late (or no) excitability, where a premature beat from an area of early repolarization can encounter a region that is still refractory, leading to the potential for re-entry if the timing and spatial characteristics align. Importantly, the excitability can be time-independent (e.g. scar is always non-excitable), or time-dependent (e.g. the R-on-T interaction is only critical when part of the tissue is already excitable again while another part is still non-excitable). These concepts illustrate how purely electrical substrate and purely structural substrate contribute to the arrhythmogenic conditions, and may potentially interact. Integrating advanced imaging techniques and computational models may help to unravel the complex mechanisms underlying arrhythmogenesis and tailor antiarrhythmic patient-specific therapy.

## Structural characterization through computer-aided imaging

The structural characteristics of the arrhythmogenic substrate do not directly reflect its electrical or arrhythmogenic properties, but they are closely interrelated. Myocardial scar can contain branching and merging bundles of surviving myocytes, causing zig-zag conduction perpendicular to myofibre direction, resulting in macroscopic slow conduction and local dispersion of excitability.^13^ Adipose tissue also leads to equivalent electrical abnormalities, with a similar propensity to VT as myocardial scar.^14^ Histology remains the gold standard for structural characterization, but is invasive, expensive, time-consuming, and offers only local information. Cardiac imaging, although offering a lower resolution than histology, provides non-invasive, valuable structural information for the entire heart.^15^

**Figure 2:**
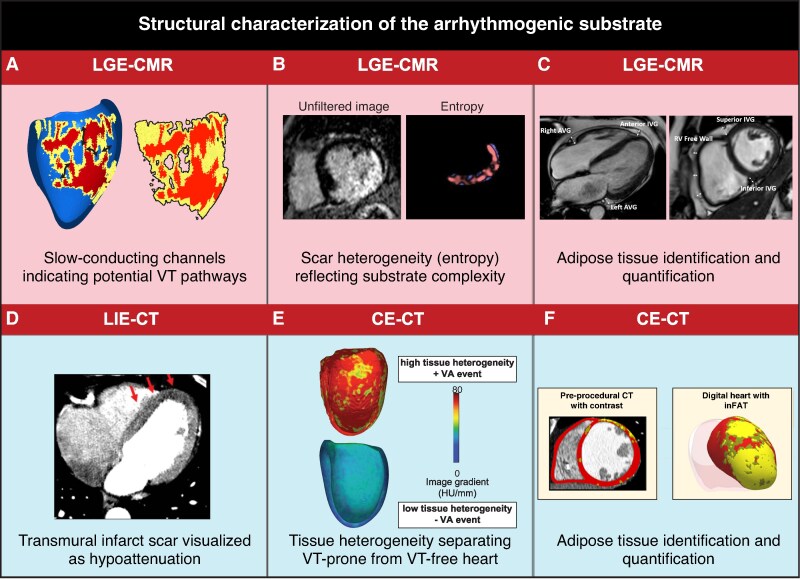
Advanced techniques for structural characterization of the arrhythmogenic substrate. (*A*) Thresholding of LGE-CMR provides a 3D model of slow-conducting channels and interface area: healthy myocardium (blue), border zone (yellow), and scar core (red) of the left ventricle, including slow conduction channels (black arrows), and the interface area between border zone and core scar.^18^ (*B*) Heterogeneity (entropy) of pixel signal intensities in LGE-CMR images, without prior thresholding.^24^ (*C*) LGE-CMR adipose tissue quantification at different locations (atrioventricular and interventricular grooves, right ventricle free wall).^22^ (*D*) Hypoattenuation of ischaemic area in dual-energy LIE-CT image.^25,26^ (*E*) Ce-CT tissue heterogeneity in ischaemic cardiomyopathy, showing a 3D model of the left ventricle in patients with high (arrhythmic) and low (arrhythmia-free) heterogeneity.^27^ (*F*) Ce-CT image and 3D model of the left ventricle divided into non-injured tissue (red), infiltrating adipose tissue (yellow), and fat–myocardium admixture (dark yellow).^28^  Ce, contrast enhanced; CMR, cardiac magnetic resonance imaging; CT, computed tomography; LGE, late gadolinium enhancement; LIE, late iodine enhancement.

*Figure 2* summarizes recent advances in cardiac imaging. Cardiac magnetic resonance imaging (CMR) facilitates comprehensive assessment of blood flow, anatomy, contractile function, and myocardial viability. The use of gadolinium contrast enhances its ability to characterize myocardial structure, with hyperenhancement in LGE-CMR indicating myocardial scarring.^16,17^ Through LGE-CMR, the myocardial wall is typically characterized into three main categories: healthy (low pixel signal intensity), border zone (intermediate intensity), and dense scar (high intensity), defined relative to a reference.^18^ The border zone is linked to slow conduction and has increased proarrhythmic potential: in patients with ischaemic cardiomyopathy, increased border zone size correlates with cardiovascular events^19^ and mortality.^20^ Specifically, corridors of border zone tissue can indicate the presence of slow-conducting channels.^18^ Identification and ablation of these channels yield superior outcomes and shorter procedural times compared to conventional VT substrate ablation.^21^ Still, the division of myocardial wall in three distinct tissue types does not fully capture the heterogeneity of scarred tissue, which may involve scar intertwined with varying degrees of surviving myofibres. Automated computational assessment of tissue heterogeneity in LGE-CMR provides more extensive details on the substrate, as it offers prognostic value for implantable cardioverter-defibrillator (ICD) therapy^22^ and cardiac mortality during follow-up.^23^

Traditional bright-blood LGE-CMR has the same high-intensity signal for scarred myocardium and blood^29^ and is typically limited to a slice thickness of ∼8 mm.^30^ Recent advances resulted in dark-blood nulling and 3D acquisition protocols, with enhanced scar-to-blood contrast resolution (+106%) and spatial resolution in all planes (1.6 mm), respectively.^29,31^ Additionally, CMR enables quantification of adipose tissue using adapted T1-weighted protocols,^32^ with CMR-detected epicardial adipose tissue thickness serving as an independent predictor of VT recurrence.^25^ Despite advancements, LGE-CMR remains technically challenging in patients with cardiac devices. Additionally, 3D CMR imaging requires longer scan times, and can be affected by frequent arrhythmias causing ghosting artefacts.^33^ Other challenges include the inability of LGE-CMR to detect diffuse fibrosis, and the lack of uniformity in defining the presence and extent of LGE.^34^

CT enables assessment of cardiac vasculature, anatomy, tissue composition, and function. Additional information can be acquired using contrast agents such as iodine (contrast-enhanced CT; Ce-CT). First-pass Ce-CT reveals segments of hypoperfusion and local wall thinning, correlating well with abnormal endocardial bipolar voltage (<1.5 mV) in ischaemic cardiomyopathy.^35^ (Ce-)CT also allows for quantification of adipose tissue,^36^ linked to VT during follow-up in both ischaemic and nonischaemic cardiomyopathy.^27^ Global left-ventricular tissue heterogeneity in Ce-CT is also associated with an increased risk of VT.^27^ While late iodine enhancement CT offers semi-quantitative analysis of scar transmurality, data on this topic are, however, scarce, and it should not be considered a viable alternative to LGE-CMR.^26^ Photon-counting CT, a relatively recent innovation, shows initial evidence of improved performance in enhancing Ce-CT’s capacity to assess adipose tissue and scar.^37^

## Electrical characterization through computer-aided invasive mapping

Invasive electrical characterization of the arrhythmogenic substrate forms the cornerstone of invasive catheter ablation for VT. This process involves detailed endocardial and sometimes epicardial mapping of the heart’s electrical activity in sinus rhythm and during VT to identify areas critical for the initiation and maintenance of VT. Targeting the substrate in scar-related VT, in addition to the clinical VT, results in better clinical outcomes.^38^

Last decades have seen a quest for the best target metric during VT ablation. Targets include: (i) local abnormal ventricular activity indicating surviving tissue bundles within scars; (ii) late potentials characterized by isolated delayed components; (iii) Purkinje potentials contributing to re-entry initiation; (iv) core isolation aiming to electrically isolate low voltage scar segments; (v) scar dechanneling targeting conducting channels within scar tissue; (vi) dynamic voltage mapping using omnipolar technology to identify border zones; (vii) decrement-evoked potentials thought to colocalize with diastolic pathways of VT circuits; and (viii) isochronal late activation mapping focusing on deceleration zones as potential focal points for re-entry. Each of these approaches aims to identify critical components of the arrhythmogenic substrate, to allow for isolation of low voltage scar segments and scar dechanneling to terminate VT.^39^

These approaches rely heavily on conventional or innovative mapping approaches. Conventional mapping approaches include activation mapping during VT, entrainment mapping to identify re-entry circuit components, pace mapping to locate VT origin or VT exit sites, and substrate mapping to define scar tissue, often using voltage criteria. However, poor haemodynamic tolerability or VT non-inducibility, along with multiple re-entry circuits within the scar, can complicate accurate isthmus delineation and targeting. Additionally, catheter ablation for scar-related VT is a demanding and lengthy procedure with significant risks^40^ and yields only modest 1-year VT-free survival rates of 30–70%.^41^ To address these challenges and enhance success rates, novel mapping approaches have been developed.

**Figure 3:**
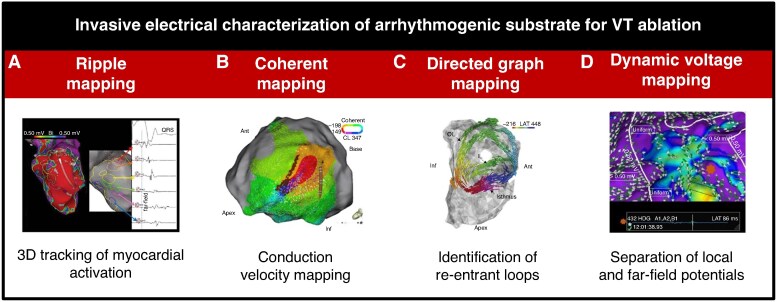
Overview of novel invasive electrical characterization mapping approaches of the arrhythmogenic substrate for VT ablation. (*A*) Ripple mapping^42^: a channel of electrograms extended from base to apex through the scar (red to purple), showing progressively delayed local activation along its course. (*B*) Coherent mapping^44^: automated conduction velocity map, indicating a basal inferolateral wall isthmus. (*C*) Directed graph mapping^45^: isthmus location of a single-loop re-entry and a possible inner-loop. (*D*) Dynamic voltage mapping^46^: changes in local voltages distinguishing uniform tissue (purple) and disarray (blue).

Recent innovative mapping techniques (*Figure 3*) include ripple mapping, coherent mapping, directed graph mapping, and dynamic voltage mapping using omnipolar technology. *Ripple mapping* is a method for three-dimensional electrogram (EGM) visualization that tracks myocardial activation.^42^ It removes the need for activation time annotation or setting a window of interest, displaying only acquired points and avoiding data interpolation. Therefore, the accuracy and reliability of the map depend heavily on point density. Ripple mapping has been shown useful to differentiate post-infarct scars from conducting border zones when superimposing ripple mapping onto a bipolar voltage map.^43^

*Coherent mapping* maps wavefront conduction speed and effectively identifies slow conduction zones during VT.^44^ It was shown that it can effectively identify slow conduction zones during VT, but that it has limited accuracy in detecting slowed conduction during substrate-based mapping.^47^ Additionally, it is critical to use three-dimensional electrical field-based approaches as traditional projections on two-dimensional mapping surfaces severely overestimate wavefront velocity.^48^ It is also important to realize that activation times cannot directly be used to determine conduction velocity in complex activation patterns that have multiple (locally independent) wavefronts, such as during sinus rhythm.

*Directed graph mapping* detects cycles in a network of nodes defined by activation times and activation directions, allowing for easy identification of re-entrant loops.^49^ This technique is rapid and showed a strong agreement with conventional activation and entrainment mapping for manually annotated VT maps.^45^ However, it tends to find a double-loop, whereas one of the circuits is in fact a passive circuit.^45,49^

*Dynamic voltage mapping using omnipolar technology* obtains orientation-independent voltage maps and automates the discrimination between low-amplitude near-field local potentials and high-amplitude far-field bystander signals. This distinction is paramount in post-MI substrate characterization, where far-field signals can mask local potentials, potentially leading to the misclassification of scar tissue as healthy myocardium. By resolving this, these algorithms increase substrate resolution and specificity. Important drawbacks include the partly subjective interpretation of vector disarray, reduced accuracy in regions with low point density, possible exclusion of true low-voltage signals due to the certainty threshold, and the occurrence of vector disarray in normal tissue, which may lead to misinterpretation.^46^

## Electrical characterization through computer-aided non-invasive mapping

Non-invasive electrical characterization covers a continuum between low- and high-resolution approaches. The traditional 12-lead electrocardiogram (ECG) can provide insight into the presence of myocardial scar through identification of poor R-wave progression over the precordial leads, the presence of Q-waves, and notching, fragmentation, and widening of the QRS complex.^50,51^ In addition, the 12-lead ECG is also used in roughly localizing the site of VT origin based on interval and morphology criteria.^52^

Presumably more accurate, higher-resolution techniques using signal-averaged ECG (SAECG) and vectorcardiography (VCG) may contribute to the detection of scar. SAECG showed delayed activation in patients with regional slow conduction,^53^ whereas VCG showed a negative correlation between lower QRS and T-wave vector magnitude and scar burden.^54^ Furthermore, a widened spatial QRS-T angle on VCG predisposes ventricular arrhythmias and cardiovascular mortality.^55^ However, contradictory data exist about the relation between VCG-derived QRS-T angle and scar burden.^54,56^

Body surface potential mapping (BSPM) has been described as an alternative to the 12-lead ECG with increased temporal and spatial resolution.^57^ BSPM has the ability to detect ischaemia^57^ but also early signs of disease in cardiomyopathies,^58^ demonstrating its potential to reveal depolarization and repolarization abnormalities that may contribute to VT/VF. Although these non-invasive techniques provide insight into the presence of electrical abnormalities, their direct relation with cardiac anatomy remains complex.

Non-invasive ‘mapping’ techniques aim to bridge from signals non-invasively recorded on the torso to signals invasively recorded directly at the myocardial surface. For example, starting from a 12-lead ECG and a patient-specific anatomy, CineECG computes the mean trajectory of the ventricular electrical activation within a 3D heart model. This enables a direct relationship between cardiac anatomy and the spatiotemporal localization of ECG waveforms.^59^ CineECG has shown to detect changes in ventricular electrical activity during induced ischaemia when changes in the ST-segment of the ECG are still subtle.^60^

Electrocardiographic imaging goes a step further and uses tens to hundreds of electrodes to obtain a BSPM, and combines that with cardiac imaging to reconstruct potentials at the cardiac surface. ECGI involves solving the ‘inverse problem of electrocardiography’, i.e. the mathematical reconstruction of local cardiac activation and repolarization patterns based on body surface ECGs.^61,62^ This inverse problem is mathematically ill-posed and ill-conditioned, which means that additional constraints have to be imposed in order to obtain a stable, realistic solution. Its development spans half a century and recent validation efforts have brought it to a level ready for scientific-clinical purposes.^61^ ECGI can localize the origin of PVCs and VT^63^ and can map re-entry circuits of induced monomorphic VTs.^64,65^ Such preprocedural mapping has helped guide non-invasive stereotactic arrhythmia radioablation (STAR).^66,67^

Next to mapping of arrhythmias, ECGI carries the potential to non-invasively characterize the electrical substrate in the absence of arrhythmias.^68–72^ Some versions of ECGI have also been validated to map repolarization times with high accuracy (*R*^2^ ≥ 0.92 with <1 cm resolution) such that it can provide insight into local regional differences in activation and repolarization times,^69,72^ although for other ECGI versions or implementations, care should be taken.^73^ Consequently, ECGI has the ability to detect steep repolarization gradients,^72^ which are known to provide a substrate for VT/VF.^68,74,75^ Other abnormal electrophysiological characteristics, such as prolonged activation-recovery intervals detected by ECGI, may also be of use for risk stratification.^76^ Furthermore, scar-related properties can be detected with ECGI through reduced amplitudes (low voltage) and fractionation in the reconstructed EGMs of post-infarct myocardium.^77^

Although ECGI has shown promising value, its implementation in clinical practice is still limited. It is challenging to validate a system that is highly sensitive to user choices (due to its ill-posed character) and its time-consuming nature (e.g. the need for model segmentation and reconstruction) disrupts clinical workflows. However, combining electrical and structural substrates non-invasively may offer unique new insights. This need for integration becomes particularly evident when considering the complementary nature of different characterization approaches.

## Electro-structural integration of modalities

Observing the arrhythmogenic substrate through different lenses—cardiac imaging, invasive mapping, and non-invasive mapping—can reveal distinct and sometimes seemingly conflicting characteristics of the underlying disease mechanisms. Each modality captures different aspects of the complex interplay between structural and electrical abnormalities that create the substrate for life-threatening arrhythmias. For example, regions showing low voltage during invasive mapping do not consistently align with areas of dense scar identified by LGE-MRI, with studies showing only ∼35% spatial overlap between these abnormalities.^78,79^ Furthermore, (proarrhythmic) heterogeneity of local repolarization duration was increased in areas with intermediate bipolar voltage amplitude in pigs after MI,^80^ but not in areas with intermediate LGE-CMR pixel signal intensity in another study.^81^ Even fewer studies have investigated the (mis)match between non-invasive mapping of scar substrate with ECGI and structural imaging.^82^ These apparent discrepancies highlight that each technique provides a unique but incomplete view of the substrate. Moreover, they may highlight our incomplete understanding of the physiological meaning of each technology’s output. Additionally, technical challenges may play a role, such as alignment mismatches between imaging and mapping systems or obtaining metrics that quantify the overlap or mismatch between structural and electrical findings.

At the same time, integration of electrical and structural modalities is already critical part of STAR as it reinforces non-invasive target delineation.^66,67^ Integration of electrical and structural characterization techniques, therefore, holds great promise for enhancing our mechanistic understanding. By combining modalities, we may better appreciate how electrical abnormalities such as conduction delay and repolarization heterogeneity interact with structural changes like scarring and fatty infiltration, to create an arrhythmogenic substrate. This integrated approach is particularly relevant as real-time integration of electrical mapping with CT and CMR-derived anatomy increasingly becomes standard practice for guiding VT ablation procedures, as highlighted in a recent consensus statement by the European Heart Rhythm Association and European Association of Cardiovascular Imaging.^83^

## Interpretation with computer simulation

While fusion of multiple modalities can provide new insights into the arrhythmogenic substrate, obtaining detailed three-dimensional mechanistic information at both high spatial and temporal resolution remains challenging with standard clinical approaches. For this purpose, patient-specific virtual heart models can act as interpretative tools.^84,85^ These models are able to simulate realistic cardiac electrophysiology with sub-second temporal resolutions within patient-specific heart and scar geometries. Accordingly, such models are used to investigate re-entrant VT circuits^86^ in more detail based on ‘zig-zag’ conduction in small channels of surviving tissue inside scar;^87^ to determine the impact of PVC location and timing on triggering VT in patient-specific scars;^84,85^ to provide critical targets for terminating VT circuits with radiofrequency ablation;^6^ to predict the efficacy of ICD therapy and post-ablation VT recurrence;^88^ and to support interpretation of optical mapping research.^89^

**Figure 4:**
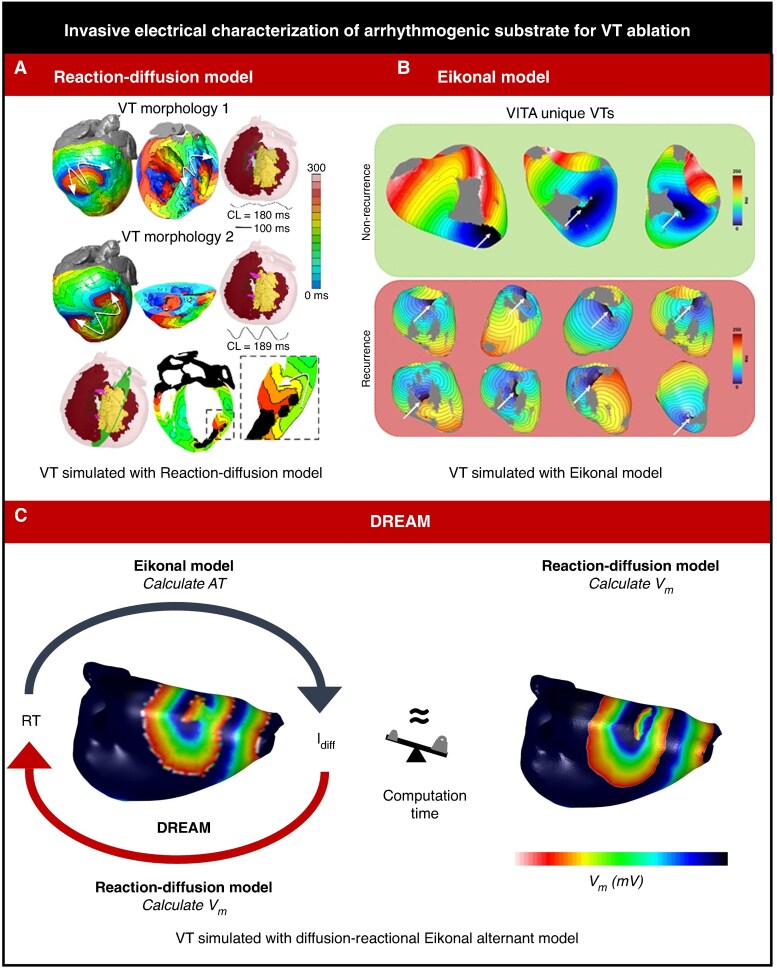
The diffusion reaction Eikonal alternant model (DREAM) combines the computationally expensive (*A*) reaction–diffusion model^99^ with the fast (*B*) Eikonal model^7^ to simulate re-entrant cardiac arrhythmias, both functional and around anatomical obstacles such as scar. (*C*) DREAM^98^ achieves faster computing times compared to monodomain simulations with RD models at mesh resolutions <0.5 mm, while maintaining accuracy even on coarse meshes at resolutions >0.5 mm where monodomain simulations can’t be performed with classic RD models. AT, activation times; *I*_diff_, diffusion current; RT, recovery times; *V*_m_, transmembrane potential voltage.

Despite such scientific advances, it remains a challenge for virtual patient-specific MI heart models to provide clinicians with valuable decision-making information within clinically realistic workflows and timeframes. Traditionally, the temporo-spatial spread of electrical activity within and around scar is described by solving reaction–diffusion equations (through non-linear partial differential equations) in a mesh of the patient-specific ventricular myocardium and scar.^90^ While robust, this is computationally expensive and time-consuming.^91^ A faster and less computationally expensive method is the reaction–Eikonal approach.^92^ This approach generates isochronal surfaces for activation wavefronts within virtual heart models using pre-defined conduction velocities,^93^ which are quick to solve on a powerful desktop computer.^94^ With this approach, however, VT simulations are only accurate over a short period of time,^95^ which consequentially neglects important interactions with repolarization dynamics and the Purkinje network.^96,97^ Recently, an alternative was developed that combines these two approaches into one, called diffusion reaction Eikonal alternant model (DREAM)^98^ (*Figure 4*). It shows promise to meet this need for speed in computer simulations of scar-mediated VT in MI patients without sacrificing accuracy. Specifically, the ability of such models to maintain accuracy at coarser mesh resolutions (<0.5 mm) is what effectively moves these simulations from offline supercomputer research to potential real-time intra-procedural tools. While these simulation approaches provide valuable mechanistic insights, they are fundamentally limited by our existing understanding of cardiac electrophysiology and the parameters we choose to include in our models.

## Unveiling hidden characteristics with artificial intelligence

Artificial intelligence offers a complementary approach that may help discover previously unrecognized patterns and relationships in cardiac data. Unlike traditional simulation methods that rely on pre-defined physiological models, AI can analyse complex datasets such as high-dimensional ECG and imaging data without predetermined assumptions about their relationships. Recent advances in this field include applications in image processing, ECG analysis, computational modelling, and workflow improvements.

Artificial intelligence can be applied in various steps of image acquisition and processing, such as segmentation, feature extraction, and classification.^100^ Extraction of classic clinical features such as the dimensions of the ventricles,^101^ and viable myocardium in LGE^102^ and non-contrast images,^103^ can help to more quickly and reliably obtain relevant clinical insights. Importantly, AI has also allowed extraction of novel image-based features, for example, for arrhythmic risk prediction.^104^

Lately, AI was extensively applied to the 12-lead ECG, for classification and novel feature extraction. Using a 12-lead ECG, AI was able to derive probabilities for a patient’s sex and body mass index, and the discrepancy between predicted and actual values was shown to provide insights into cardiac disease.^105^ Combined with clinical information, such models could also be used to screen for subtle disease progression in cardiomyopathies,^106^ identify individuals at risk of developing ventricular arrythmias,^107^ and more generally major adverse cardiovascular events.^108^ It is also possible to combine multiple modalities, e.g. integrating cardiac imaging with ECG to predict sudden cardiac death.^109^ However, only a limited number of such multi-modal models have been prospectively evaluated in clinical practice.^110,111^ Other challenges with AI include their need for large, high-quality datasets, inherent bias, limited generalization potential, and interpretation challenges (‘black box’).

AI also plays a role in computational modelling, where phenomena are generally understood well enough to be captured in precise biophysics simulations, but may still benefit from AI integration. For example, AI allows augmentation of the well-defined physics of the inverse ECGI reconstruction process with information such as the number of leads, a patient-specific geometry, physical constraints, and other patient-specific physiological information that was obtained through other modalities.^112,113^ Compared to traditional methods that rely on biophysical assumptions, AI techniques provide the flexibility to train on various relevant metrics by using a sufficiently large neural network, and may be more robust to noise.^114^

Despite these great promises for research, AI’s largest initial impact may be on workflow improvement. For example, it has been used to optimize invasive mapping procedures, integrate information from AI-based imaging analysis,^115^ and obtain the activation sequence by reconstructing EGMs.^114^ Furthermore, AI could streamline ECGI’s application in a clinical setting by addressing its labour-intensive and time-consuming elements. For example, the patient-specific geometry typically required for ECGI could be omitted by using a 2D standardized representation.^116^ This would remove the need for imaging acquisition and image processing, and its improved clinical utility may offset the potential reduction in accuracy. Although these workflow improvements do not reveal substrate characteristics on their own, they facilitate data processing at a larger scale, enabling the extensive studies necessary to investigate and validate novel insights.

These AI-driven approaches have the potential to enhance our understanding of arrhythmogenic substrates, improve risk stratification, and guide personalized treatment strategies.^89,117^ However, the successful integration of AI into mechanistic research and routine clinical care will require careful validation, consideration of interpretability, and ongoing evaluation of its impact on patient outcomes.^118–120^

## Future perspective

Looking ahead, we can envision how the advanced computer-aided techniques described throughout this review, which are summarized for their primary application, clinical value and research value in *Table 1*, will continue to improve our understanding, localization, and characterization of arrhythmogenic substrates, whereas challenges remain on the path to actual clinical impact. Post-MI VT serves as an ideal model to study how computational approaches can enhance clinical practice because the arrhythmogenic mechanisms underlying these VTs are relatively well-known and these VTs depend on a critical interaction between structural factors (surviving myocardium in scar tissue, adipose tissue) and functional factors (delayed activation, slowed conduction, current-to-load mismatch, repolarization gradients).^121,122^ The lessons learned from this domain will likely extend to other arrhythmic conditions with more complex or less understood mechanisms.

**Table 1:** Computer-aided technologies for arrhythmogenic substrate characterization

Specific technique	Primary application	Clinical value	Research value
**Structural imaging**			
Dark-blood LGE-CMR	Scar border zone delineation	Improved contrast, channel identification for targeted ablation	Validation of electrical-structural relationships
3D acquisition CMR	High-resolution spatial imaging	Enhanced spatial resolution in all planes, comprehensive substrate visualization	Detailed 3D substrate reconstruction for modelling
Tissue heterogeneity (mean entropy)	Risk stratification	ICD therapy prediction, mortality assessment	Novel biomarker development
Photon-counting CT	Adipose tissue assessment	VT recurrence prediction	Fat infiltration mechanisms in arrhythmogenesis
Wall thickness CT	Structural substrate mapping	Correlation with voltage abnormalities, hypoperfusion detection	Substrate characterization in non-MRI candidates
**Invasive mapping**			
Omnipolar/dynamic voltage	Border zone identification	Individualized substrate characterization	Viable vs. non-viable tissue discrimination
Coherent mapping	Conduction velocity assessment	Slow conduction zone identification during VT	3D wavefront velocity validation
Directed graph mapping	Re-entry circuit detection	Rapid identification, reduced procedure time	Automated circuit topology analysis
Ripple mapping	Scar-border differentiation	No manual annotation needed	Unbiased activation pattern visualization
**Non-invasive mapping**			
ECGI	Pre-procedural planning	STAR guidance, VT localization	Inverse problem methodology advancement
CineECG	Cardiac electrical activity trajectory	Anatomical correlation of ECG	Simplified ECGI alternative development
BSPM	Substrate detection	Early disease detection	High-resolution surface potential patterns
**Personalized computer simulations**			
Virtual heart models	Ablation target identification	Personalized therapy planning	Mechanistic insights into re-entry
DREAM simulation	VT mechanism prediction	Fast patient-specific modelling	Computational efficiency optimization
**AI applications**			
Deep learning ECG	Risk stratification	SCD prediction, cardiomyopathy screening	Hidden feature discovery
Image-based AI	Feature extraction	Automated scar analysis	Pattern recognition in complex substrates
Multi-modal AI	Integrated assessment	Combined ECG & imaging risk prediction	Cross-modality relationship discovery

AI, artificial intelligence; BSPM, body surface potential mapping; CMR, cardiac magnetic resonance imaging; CT, computed tomography; DREAM, diffusion reaction Eikonal alternant model; ECG, electrocardiogram; ECGI, electrocardiographic imaging; ICD, implantable cardioverter-defibrillator; LGE-CMR, late gadolinium enhancement cardiac magnetic resonance imaging; SCD, sudden cardiac death; STAR, stereotactic arrhythmia radio ablation; VT, ventricular tachycardia.

### Technological developments

Future increases in resolution of imaging and inverse electrocardiographic techniques will allow for a more thorough non-invasive *in vivo* characterization of the structural and functional arrhythmogenic substrate. Subsequent integration of electrical and structural characteristics of an individual’s heart may bring new insights into the risk of arrhythmia initiation, and allow for patient-specific interpretation of multimodal data through personalized computer simulations. Such technological improvements are critical to deal with the growing complexity of the underlying data, which makes manual analysis more challenging. This requires further development of approaches that help streamline the creation of such personalized models from patient data, and their efficient evaluation.

Advancements in AI, particularly deep learning models trained on extensive datasets of clinical data, ECG and imaging, may facilitate automated feature extraction and enhance risk prediction. Techniques such as transfer learning and few-shot learning could enable AI models to adjust to new patient populations or arrhythmia types with limited data.^123^ The hybrid integration of AI with mechanistic computational models may yield more interpretable, generalizable, and verifiable models for arrhythmia characterization and risk stratification.

### Clinical applications

Technical improvements are essential to strengthen the value of computer-aided tools personalized medicine. Integrating imaging-derived scar maps with ECGI-based electrical substrate characterization could guide personalized ablation strategies, potentially reducing procedure times and enhancing success rates. AI algorithms that analyse ECG and clinical data may identify high-risk patients who would benefit from prophylactic ICD implantation or more intensive follow-up. Patient-specific simulations could predict the optimal anti-arrhythmic drug regimen or help optimize cardiac resynchronization therapy settings. Virtual-reality systems that incorporate patient-specific anatomy and electrophysiology could improve physician training and treatment planning. Patient-specific simulation may become a standard part of care for various arrhythmic syndromes.

### Translation towards other arrhythmic syndromes

Computer-aided techniques can also be applied to other arrhythmic syndromes. In cardiomyopathy-related arrhythmias, the structural alterations are probably more diffuse than in the post-MI VTs, and the need for an integrative electro-structural perspective is even more urgent. For example, the J-wave syndromes (Brugada Syndrome, the Early Repolarization Syndrome)^124,125^ were initially considered purely functional, but have recently been described as a subepicardial cardiomyopathy when subtle structural abnormalities were observed in the subepicardial myocardium.^124,126^ The critical interplay between localized (structural) fibrosis and (functional) conduction failure may lie at the basis of both the arrhythmias and the electrocardiographic signs associated with these syndromes.^127^

Advanced multi-modal imaging, high-resolution mapping, and computational modelling may provide insights into the mechanisms by which non-myocyte cells—such as fibroblasts, macrophages, and fatty infiltrations—influence arrhythmia susceptibility through effects on tissue heterogeneity and electrophysiological remodelling in the ventricles.^128^ Similar approaches may also apply to atrial tachy-arrhythmias and atrial fibrillation, where a similar interaction between structural and functional arrhythmogenic conditions applies.^129,130^

### Challenges and future directions

The computational demands of high-resolution substrate characterization, resource-intensive simulations, and the need for large, diverse datasets for AI model development bring significant challenges. Standardization of data acquisition and annotation protocols will be essential for the generalizability and interoperability of computer-aided techniques. Health-economic studies will be required to demonstrate the cost-effectiveness and clinical utility of these approaches in a clinical setting. Importantly, as computer-aided techniques become more sophisticated, it will be crucial to maintain interpretability and avoid over-reliance on ‘black box’ models. The goal should be to augment, rather than replace, clinical decision-making.

## Conclusion

The future of arrhythmia management and research lies in the integration of advanced computer-aided techniques with clinical expertise. As we outlined, technological developments in high-resolution imaging, inverse electrocardiography, computational modelling, and AI are critical to enhance our understanding of arrhythmogenic substrates. These advancements may yield novel scientific insights into the interplay between structural and functional factors in various arrhythmic syndromes, varying from post-MI VTs to rare channelopathies. In clinical practice, these tools have the potential to enable highly automated, patient-specific risk stratification, diagnosis, and treatment planning for potentially lethal ventricular arrhythmias. However, realizing this potential will require overcoming significant challenges in data standardization, computational efficiency, and clinical validation. Finally, a heart is more than a collection of myocytes and fibrocytes. Future research must account for the modulating effects of neurons, inflammatory cells, and adipocytes on arrhythmogenic substrates. By embracing these computer-aided approaches and benefiting from their integration, we can maintain a holistic view of the complexities of cardiac (patho-)physiology and work towards a future where personalized, precise management of complex arrhythmias becomes a reality.

## Data Availability

No new data were generated or analysed in support of this research.
